# Causal association between phenylalanine and Parkinson’s disease: a two-sample bidirectional mendelian randomization study

**DOI:** 10.3389/fgene.2024.1322551

**Published:** 2024-07-01

**Authors:** Shiqing Li, Huangcheng Song, Cong Yu

**Affiliations:** Nantong Haimen People’s Hospital, Neurosurgery Department, Jiangsu, China

**Keywords:** phenylalanine, Parkinson’s disease, causal relationship, mendelian randomization, genome-wide association study

## Abstract

**Background:**

Research findings indicate a putative indirect or latent association between phenylalanine (Phe) and Parkinson’s disease (PD). In this study, we aimed to analyze the causal relationship between Phe and PD by two sample Mendelian randomization (MR) analysis.

**Methods:**

In this study, the PD-related dataset and Phe-related dataset were downloaded from Integrative Epidemiology U1nit (IEU) Open Genome-Wide Association Study (GWAS) database. Four algorithms (MR Egger, maximum likelihood, inverse variance weighting (IVW) and unweighted regression) were used to perform MR analysis. The sensitivity analysis (heterogeneity test, horizontal pleiotropy test and Leave-One-Out (LOO) analysis) was used to assess the reliability of MR analyses.

**Results:**

In the forward MR analysis, Phe was a safety factor for PD (*p*-value < 0.05 and odds ratios (OR) < 1). The results of reverse MR analysis showed that there was no causal relationship between PD and Phe (*p*-value > 0.05). In addition, sensitivity analysis showed that MR analysis was reliable.

**Conclusion:**

The results of this study revealed that Phe was a safety factor for PD, meaning that Phe reduced the risk of PD.

## 1 Introduction

Parkinson’s disease (PD) is a common type of central nervous system disease which occurs mostly in middle-aged and elderly people. It is characterized by the loss of dopaminergic neurons in the nigrostriatal pathway and the formation of lewy bodies (Parmar et al., 2020). When more than 50% of dopaminergic neurons are lost, the patient experience typical motor symptoms (Armstrong and Okun, 2020), including resting tremor, muscle stiffness, slow movement, and may be accompanied by mental disorders, dementia, and memory problems. PD has a high disability rate, and patients will gradually lose their self-care ability and eventually become bedridden. Being bedridden can cause a variety of complications and is closely related to the mortality of PD (Olanow and Tatton, 1999; Fabbri et al., 2022). According to the report, there were 1,081.72×10^3^ cases worldwide in 2019 with an overall age-standardized incidence that has significantly increased (Ou et al., 2021).The pathogenesis of PD is not fully understood. However, many studies have found that the possible pathogenesis of PD includes protein processing defects, mitochondrial dysfunction, oxidative stress and inflammation (Büeler, 2010; Zuo and Motherwell, 2013; Camilleri and Vassallo, 2014; Celardo et al., 2014). At present, there is no complete cure for PD, which can only relieve symptoms (Simon et al., 2020).

Studies have shown that increasing dopamine concentration in the striatum is an effective treatment strategy for PD (Cools, 2006; Li et al., 2022). The current effective drug treatment involves taking levodopa (3.4- dihydroxyl-Phe, L-DOPA), a precursor of dopamine. L-DOPA can be converted into dopamine, which regulates neuronal activity in the striatum. In addition, the striatum is the main target of dopaminergic neurons. Dopamine is synthesized from tyrosine in dopaminergic neurons. Phenylalanine (Phe) can provide dopamine to the brain through the Phe-tyrosine-dopa-dopamine pathway. These results may reflect that Phe might be related to PD.

Mendelian randomization (MR) is a method that uses instrumental variables (IVs) to explore the causal relationship between exposure factors and outcomes. The screening of IVs should be closely related to exposure factors and independent of outcome, and IVs is after single nucleotide polymorphism (SNP) screening. A reliable MR analysis should satisfy the following conditions. Firstly, IVs is strongly associated with exposure and not with outcome, Secondly, IVs should not be associated with potential confounders. Finally, IVs can only affect the outcome through exposure factors.

Two-sample MR analysis uses genetic data from two samples of the same population for analysis. Bidirectional MR analysis assesses whether there is reverse causality between exposure factor and outcome. The method is to perform two-sample MR analyses twice, and the SNPs used in these two analyses should be completely different. According to our limited knowledge, there is currently a lack of studies to explore the two-way causal relationship between Phe and PD.

Based on the Genome-wide association study (GWAS), the causal relationship between Phe and PD was evaluated by two-way MR analysis, and sensitivity analysis was performed to ensure the reliability of the MR analysis results. This study provides a new reference for the causal relationship between Phe and PD, and provide new insights for the treatment of PD.

## 2 Materials and methods

### 2.1 Data source and pre-processing

Our study was a bidirectional two-sample MR analysis. The Integrative Epidemiology Unit (IEU) Open GWAS database (https://gwas.mrcieu.ac.uk/) was utilized to download Parkinson’s disease -related dataset (finn-b-G6_PARKINSO) and Phenylalanine (Phe)-related dataset (met-c-919). The finn-b-G6_PARKINSO contained 218,792 samples and 16,380,461 SNPs. The met-c-919 contained 22,663 samples and 12,042,964 SNPs. The R package “TwoSampleMR” was used to read exposure factors and screen SNPs (*p* < 5 * 10–6), and linkage disequilibrium analysis (LDA) were performed to ensure independence (r2 = 0.001 and kb = 10,000).

### 2.2 Mendelian randomization analysis

In forward MR analysis, the exposure factor was Phe and the outcome was PD. In reverse MR analysis, the exposure factor was PD and the outcome was Phe. The “mr” function was combined with four algorithms to carry out MR analysis (MR Egger, Maximum likelihood, Inverse variance weighted (IVW), Unweighted regression). Among various methods, the most important method was the IVW. Then, odds ratios (OR) were calculated, OR value greater than one indicated that exposure factor was risk factor, OR less than one indicated that exposure factor was safety factor.

### 2.3 Sensitivity analysis

The sensitivity analysis was conducted *via* heterogeneity test, the horizontal pleiotropy test and the Leave-One-Out (LOO) analysis. If the Q_pval of the heterogeneity test was greater than 0.05, indicating there was no heterogeneity. The *p*-value of horizontal pleiotropy was greater than 0.05, indicating no horizontal pleiotropy. LOO was used to observe the effect of removing a SNP on the result.

### 2.4 Ethics approval and consent to participate

This study utilized summary-level data from published studies and publicly available GWASs, with ethical approvals obtained from the respective institutional review boards, and informed consent was provided by all participants involved in the original studies.

## 3 Results

### 3.1 Causal effect of phe on PD in forward mendelian randomization analysis

**FIGURE 1:**
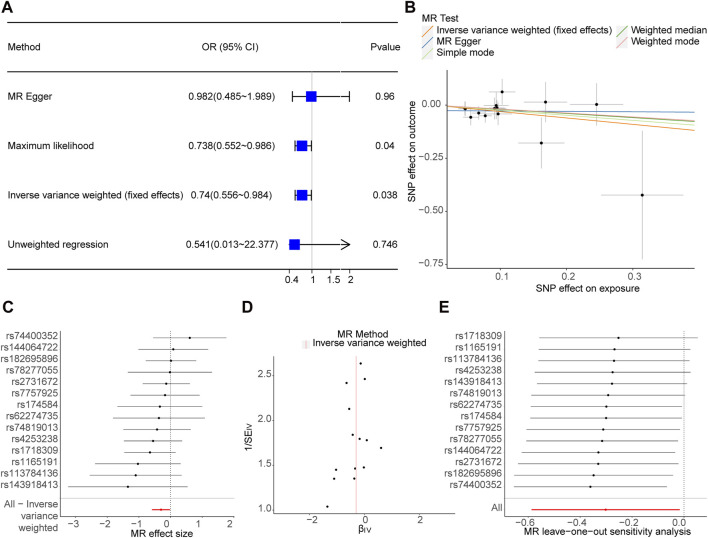
Causal effect of phenylalanine (Phe) on Parkinson’s disease (PD) in forward Mendelian randomization (MR) analysis. **(A)** Estimation of associations between Phe and risk of PD using MR analysis. **(B)** Scatter plot of genetic associations between exposure and outcome. Phe and PD. **(C)** Display of the forest plot for the single nucleotide polymorphism (SNP) analysis of Phe increasing on PD risk. The *x*-axis shows the MR effect size for Phe increasing on PD, while the y-axis illustrates the analysis for each of the SNPs. The dot and bar indicate the causal estimate and 95% confidence intervals (CI) of the association between Phe increasing and PD risk. **(D)** Phe-PD funnel plot. It is found that based on inverse variance weighted (IVW) method, almost left-right symmetry. **(E)** Presentation of the leave-one-out sensitivity analysis for the effect of Phe increasing SNPs on PD risk in the context of MR. The dot and bar indicate the estimate and 95% CI when a specific SNP is removed. OR, odds ratios; IV, instrumental variant; SE, standard error.

After screening, 14 SNPs related to Phe and unrelated to PD were obtained (Supplementary Table S1). The results of the IVW (*p*-value = 0.038 and OR = 0.740) and Maximum likelihood (*p*-value = 0.040 and OR = 0.738) MR algorithms indicated that Phe was a safety factor for PD (Figure 1A; Table 1). The slope of the lines in the scatter plot was negative, indicating that Phe levels would reduce the risk of PD (Figure 1B). The IVW algorithm that the effect values of Phe on PD, indicating that the Phe were safety factors on the PD (Figure 1C). In addition, the samples were symmetrically distributed along both sides of the IVW line in the funnel plot, indicating that the MR analysis conformed to randomness (Figure 1D).

**TABLE 1:** Mendelian randomization assessment of Phenylalanine causal influence on Parkinson’s disease.

Exposure	Outcome	Method	*p*-value	OR	OR_lci95	OR_uci95
Phenylalanine	Parkinson’s disease	MR Egger	0.96	0.982	0.485	1.989
		Maximum likelihood	0.04	0.738	0.552	0.986
		Inverse variance weighted (fixed effects)	0.038	0.74	0.556	0.984
		Unweighted regression	0.746	0.541	0.013	22.377

In order to judge the reliability of the analysis results, sensitivity analysis was carried out. The heterogeneity test results of the two algorithms showed that Q_pval of MR Egger (Q_pval = 0.811) and IVW (Q_pval = 0.817) were both greater than 0.05, indicating no heterogeneity of MR analysis (Table 2). According to horizontal pleiotropy test, *p*-value was greater than 0.05 (*p*-value = 0.407), indicating no horizontal pleiotropy of results (Table 3). In addition, the LOO results showed that the MR results were reliable (Figure 1E).

**TABLE 2:** The heterogeneity test results of Phenylalanine causal influence on Parkinson’s disease.

Exposure	Outcome	Method	Q	Q_df	Q_pval
Phenylalanine	Parkinson’s disease	MR Egger	7.655	12	0.811
		Inverse variance weighted	8.394	13	0.817

**TABLE 3:** The horizontal pleiotropy results of Phenylalanine causal influence on Parkinson’s disease.

Exposure	Outcome	Egger_intercept	SE	*p*-value
Phenylalanine	Parkinson’s disease	−0.026	0.03	0.407

### 3.2 No causal effect of PD on phe in reverse mendelian randomization analysis

**FIGURE 2:**
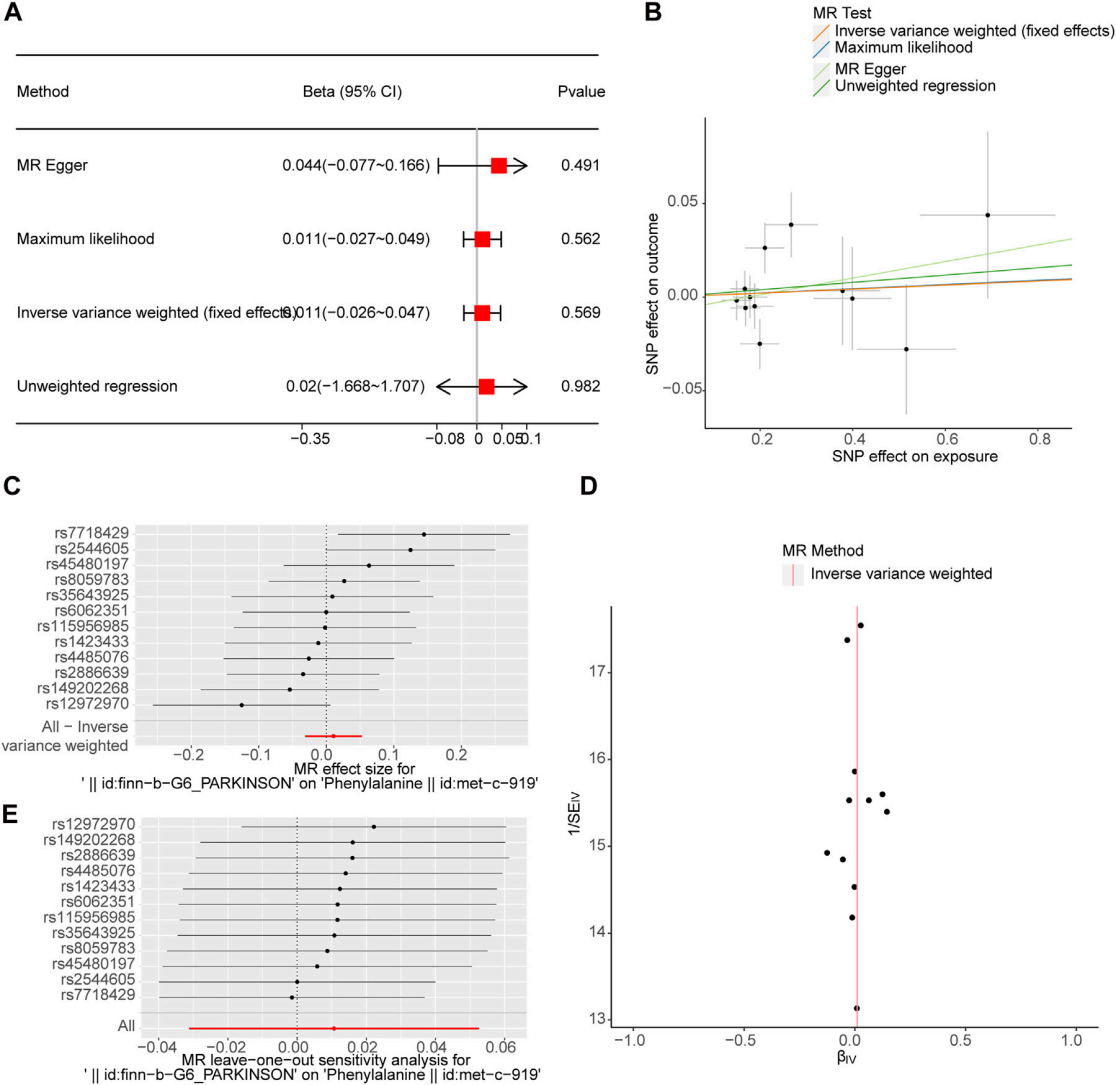
No causal effect of PD on Phe in reverse MR Analysis. **(A)** Estimation of associations between PD and risk of Phe using MR analysis. **(B)** Scatter plot of genetic associations between exposure and outcome. PD and Phe. **(C)** Display of the forest plot for the single SNP analysis of PD increasing on Phe risk. **(D)** PD-Phe funnel plot. It is found that based on IVW method, almost left-right symmetry. **(E)** Presentation of the leave-one-out sensitivity analysis for the effect of PD increasing SNPs on Phe risk in the context of MR.

After screening, 12 SNPs related to PD and unrelated to Phe were obtained (Supplementary Table S2). The *p*-value of the four MR methods were all greater than 0.05, indicating that there was no causal relationship between PD and Phe (Figure 2A; Table 4). The combination of forest plots, scatter plots and funnel plots supported that there was no causal relationship between PD and Phe (Figures 2B–D). The results of sensitivity analysis showed that the reverse MR analysis was reliable (Table 5, 6; Figure 2E).

**TABLE 4:** Mendelian randomization assessment of Parkinson’s disease causal influence on Phenylalanine.

Exposure	Outcome	Method	*p*-value	Beta	Beta_lci95	Beta_uci95
Parkinson’s disease	Phenylalanine	MR Egger	0.491	0.044	0.1656	−0.0771
		Maximum likelihood	0.562	0.011	0.0488	−0.0265
		Inverse variance weighted (fixed effects)	0.569	0.011	0.0474	−0.026
		Unweighted regression	0.982	0.02	1.7074	−1.6684

**TABLE 5:** The heterogeneity test results of Parkinson’s disease causal influence on Phenylalanine.

Exposure	Outcome	Method	Q	Q_df	Q_*p*-value
Parkinson’s disease	Phenylalanine	MR Egger	13.839	10	0.18
		Inverse variance weighted	14.306	11	0.217

**TABLE 6:** The horizontal pleiotropy results of Parkinson’s disease causal influence on Phenylalanine.

Exposure	Outcome	Egger_intercept	SE	*p*-value
Parkinson’s disease	Phenylalanine	−0.008	0.013	0.574

## 4 Discussion

PD is the second most common neurodegenerative disease (Ou et al., 2021). Its main pathological features include the loss of dopaminergic neurons in the nigro-striatum pathway and the formation of lewy bodies. PD seriously affects the quality of life of patients and can lead to catastrophic complications. Therefore, it is crucial to deepen the understanding of the pathogenesis of PD and guide its treatment. Tyrosine is converted to L-DOPA by the action of tyrosine hydroxylase, and then L-DOPA is converted to dopamine by the action of aromatic amino acid decarboxylase. Dopa serves as precursor of dopamine, norepinephrine and epinephrine. Phe can be converted into tyrosine by the action of phenylalanine hydroxylase. Therefore, we speculate that Phe may be related to PD. This study aims to use MR analysis to investigate the potential causal relationship between Phe and PD. By analyzing relevant datasets and utilizing different MR algorithms, we seek to understand the impact of Phe on PD. These findings are significant as they can provide insights into the role of Phe in PD and inform potential therapeutic strategies targeting Phe metabolism in the context of PD.

Phe is both an aromatic amino acid and an essential amino acid. Current researches on the relationship between Phe and PD seems to be more inclined to use Phe as a biomarker for the diagnosis of PD. A study published in 2016 compared specimens from 42 PD patients and 16 control subjects, as well as serum specimens from 28 newly diagnosed PD patients, 52 PD patients treated with L-DOPA, and 27 control subjects (Hirayama et al., 2016).The researchers found that serum Phe was high in newly diagnosed patients but not in treated patients. Tyrosine/Phe ratios were lower in both of these two groups. Another study involving 100 PD patients and 50 control subjects performed a metabolomic analysis of urine samples using high-resolution nuclear magnetic resonance spectroscopy (Kumari et al., 2020a). This study found that Phe in the urine of PD patients was significantly increased. Previous research has also shown that the Phe concentration in the cerebrospinal fluid of PD patients is reduced, while the concentration in feces and saliva is increased (Öhman and Forsgren, 2015; Kumari et al., 2020b; Vascellari et al., 2020). However, a 2020 meta-analysis found no abnormalities of Phe in cerebrospinal fluid and blood (Jiménez-Jiménez et al., 2020). These studies only observed biological phenomena without revealing mechanisms or clarifying causal relationships between Phe and PD. In fact, it is possible that the onset of PD in turn affects the changes in these measures. For instance, PD patients consume more energy in the resting state than healthy people, and this increase in consumption may cause metabolic changes (Toth et al., 1997). In addition, the physiological changes related to PD and the influence of treatment can also cause changes in the amino acid content of the human body. These factors include competitive absorption with levodopa drugs, altered hormone concentrations (Jurado-Coronel et al., 2018), endothelial dysfunction (Yoon et al., 2014), and impaired muscle quantity or quality (da Luz et al., 2021).

In this study, the MR analysis was used to explore the relationship between Phe and PD. Firstly, we downloaded the data set and used the same standard (*p* < 5*106, clump = TRUE; r2 = 0.001; kb = 10,000) filtering IVs. In Table 1, F-values of all SNPS are greater than 10, indicating that bias caused by weak instrumental variables can be effectively avoided. Then forward MR analysis was performed to analyze the effect of Phe on PD, in which the exposure factor was Phe, the outcome was PD. It was found that Phe is a protective factor for PD. In addition, we also used sensitivity analysis to further ensure the reliability of MR analysis. Finally, we also conducted a reverse MR analysis to eliminate the reverse causal effect in this study and ensure the reliability of the conclusion. Previous studies have suggested that damage to the blood-brain barrier function in the pathological progression of PD can further promote the development of PD (Liu et al., 2023). Additionally, research has also indicated that aromatic amino acids, such as phe, can compete with branched-chain amino acids to penetrate the blood-brain barrier (She et al., 2023). The increase in aromatic amino acids leads to the formation of false neurotransmitters, which in turn inhibit the central nervous system. These findings suggest that a potential mechanism involving phenylalanine and PD may be related to their competition for entry into the brain through the blood-brain barrier and the subsequent impact on neurotransmitter activity in the central nervous system. Furthermore, research has also found that phenylalanine consumption can reduce the biosynthesis of dopamine (Xu et al., 2024). The main characteristic of Parkinson’s disease is the reduction or loss of dopaminergic neurons in the substantia nigra (SN) (Liu et al., 2023). These findings suggest that a potential mechanism involving phenylalanine and Parkinson’s disease may be related to the impact of phenylalanine on dopamine biosynthesis, which is crucial for the function of dopaminergic neurons in the SN.

So far, some studies have suggested that amino acids may have an effect on the development of neurodegenerative diseases (Tsai and Coyle, 1995; Seshadri et al., 2002; Bycroft et al., 2018; Correale, 2020). Building on this, Cheng et al., (2023) used MR analysis to analyze the potential causality of nine amino acids and six neurodegenerative diseases. Their study found that glutamine is a safety factor in Alzheimer’s disease, while leucine is a protective factor in PD. However, their study did not find a causal association between Phe and PD. The possible reason is that their study chose a different data set from ours, and chose different IVs, which may have had an impact on the results.

MR analysis supported a significant causal relationship between Phe and PD, while did not reveal the mechanism of action of Phe in PD. Therefore, we should be cautious about the biological revelation of this conclusion. Although Phe is the earliest studied amino acid associated with PD, there are currently no firm conclusions about its effect on the development of PD. Some scholars described that Phe absorption in PD patients is impaired (Braham et al., 1969). However, other scholars found that the absorption of this amino acid is not affected (Granerus et al., 1971). Thus, there is no clear reason to supplement exogenous phenylalanine (Earp et al., 2023). Yan et al. identified gut metabolites associated with PD through bioinformatics analysis (Yan et al., 2022).They found that increased Phe in feces was a risk factor of environmental exposure, and it was involved in the development of PD through four pathways, such as “apoptosis signaling pathway” and “inflammation mediated by chemokine and cytokine signaling pathway”, *etc.* However, for the change of Phe in the feces of PD patients, there was a totally different report (Yan et al., 2021). As the disease progresses, Phe in the patient’s blood decreases (Figura et al., 2018; Zhang et al., 2022). The above research may reveal that Phe plays a role in the development of the disease. In conclusion, the action mechanism of Phe in PD is still unclear, and more studies are needed to explore it.

The advantage of this study lies in the use of MR for causal inference, which avoids the influence of confounding factors. Additionally, the reverse MR analysis was conducted to avoid the bias caused by reverse causation. However, the weakness of our study is that the selected study population is European and may not be representative of the entire population. Furthermore, MR analysis cannot reveal the mechanism of Phe’s influence on PD. In summary, we found the causal relationship between Phe and PD through bidirectional MR analysis, providing a reference for clinical decision-making. Therefore, it is necessary to include larger sample sizes from both European and non-European populations in further MR analyses to ensure broader applicability of our conclusions. Furthermore, as we did not demonstrate correlations between PD stage, disease duration, or time of onset, our future research will aim to further investigate and clarify the mechanisms and causal relationships between Phe and PD, taking into consideration factors such as PD stage, disease duration, and time of onset. Additionally, to address the limitation of our study regarding the lack of a control group, we plan to incorporate one in future research to enhance the comprehensiveness and reliability of our comparative data.

## Data Availability

The original contributions presented in the study are included in the article/Supplementary Material, further inquiries can be directed to the corresponding author.

## Abbreviations

Phe, phenylalanine; PD, Parkinson’s disease; MR, Mendelian randomization; IVW, inverse variance weighting; LOO, Leave-One-Out; IEU, Integrative Epidemiology Unit; GWAS, Genome-Wide Association Study; OR, odds ratios; IVs, instrumental variables; SNP, single nucleotide polymorphism; LDA, linkage disequilibrium analysis; CI, confidence intervals; SE, standard error.
